# In vivo validation of a miniaturized electrochemical oxygen sensor for measuring intestinal oxygen tension

**DOI:** 10.1152/ajpgi.00050.2019

**Published:** 2019-06-12

**Authors:** Mark E. Gray, Jamie R. K. Marland, Camelia Dunare, Ewen O. Blair, James Meehan, Andreas Tsiamis, Ian H. Kunkler, Alan F. Murray, David Argyle, Alex Dyson, Mervyn Singer, Mark A. Potter

**Affiliations:** ^1^The Royal (Dick) School of Veterinary Studies and Roslin Institute, University of Edinburgh, Edinburgh, United Kingdom; ^2^Cancer Research United Kingdom Edinburgh Centre and Division of Pathology Laboratories, Institute of Genetics and Molecular Medicine, University of Edinburgh, Edinburgh, United Kingdom; ^3^School of Engineering, Institute for Integrated Micro and Nano Systems, Edinburgh, United Kingdom; ^4^Bloomsbury Institute of Intensive Care Medicine, Division of Medicine, University College London, London, United Kingdom; ^5^Department of Surgery, Western General Hospital, Edinburgh, United Kingdom

**Keywords:** hypoxemia, intestinal resection and anastomosis, intestinal serosal tissue oxygen tension, miniaturized electrochemical oxygen sensor, superior mesenteric artery occlusion

## Abstract

Recent advances in the fields of electronics and microfabrication techniques have led to the development of implantable medical devices for use within the field of precision medicine. Monitoring visceral surface tissue O_2_ tension ($\text{P}{\text{T}}_{{\text{o}}_{2}}$) by means of an implantable sensor is potentially useful in many clinical situations, including the perioperative management of patients undergoing intestinal resection and anastomosis. This concept could provide a means by which treatment could be tailored to individual patients. This study describes the in vivo validation of a novel, miniaturized electrochemical O_2_ sensor to provide real-time data on intestinal $\text{P}{\text{T}}_{{\text{o}}_{2}}$. A single O_2_ sensor was placed onto the serosal surface of the small intestine of anesthetized rats that were exposed to ischemic (superior mesenteric artery occlusion) and hypoxemic (alterations in inspired fractional O_2_ concentrations) insults. Control experiments demonstrated that the sensors can function and remain stable in an in vivo environment. Intestinal $\text{P}{\text{T}}_{{\text{o}}_{2}}$ decreased following superior mesenteric artery occlusion and with reductions in inspired O_2_ concentrations. These results were reversible after reinstating blood flow or by increasing inspired O_2_ concentrations. We have successfully developed an anesthetized rat intestinal ischemic and hypoxic model for validation of a miniaturized O_2_ sensor to provide real-time measurement of intestinal $\text{P}{\text{T}}_{{\text{o}}_{2}}$. Our results support further validation of the sensors in physiological conditions using a large animal model to provide evidence of their use in clinical applications where monitoring visceral surface tissue O_2_ tension is important.

**NEW & NOTEWORTHY** This is the first report of real-time continuous measurements of intestinal oxygen tension made using a microfabricated O_2_ sensor. Using a developed rodent model, we have validated this sensor's ability to accurately measure dynamic and reversible changes in intestinal oxygenation that occur through ischemic and hypoxemic insults. Continuous monitoring of local intestinal oxygenation could have value in the postoperative monitoring of patients having undergone intestinal surgery.

## Graphical abstract

**Figure:**
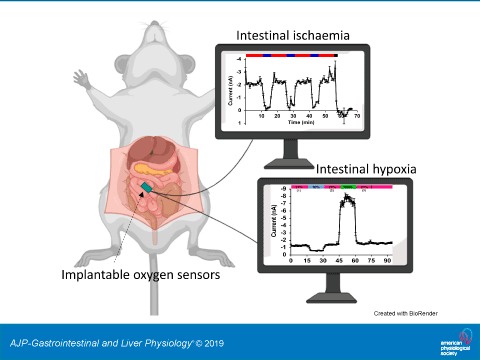

## INTRODUCTION

Precision medicine is beginning to play important roles in the prevention, investigation, and treatment of a range of diseases, as it enables individual patient variability to be considered. Precision medicine may be integrated into standard clinical practices in different ways; however, recent advances in the fields of electronics and microfabrication techniques have led to interest in the use of implantable medical devices for disease diagnosis, treatment, and monitoring. Preclinical research using in vivo murine studies have already shown that implantable devices can detect tumor-secreted biomarkers (8) or release chemotherapeutic drugs directly within tumors (29). The biocompatibility and biofunctionality of implantable devices in a range of diseases have also been shown in many studies using in vivo models, providing substantial evidence of their increasing potential for clinical applications (15).

One such implantable medical device is being developed through the Implantable Microsystems for Personalized Anti-Cancer Therapy (IMPACT) program at the University of Edinburgh (27). This project has developed a novel, miniaturized implantable O_2_ sensor on a silicon chip designed to monitor intratumoral O_2_ levels. The design is based on a Clark electrode for the electrochemical detection of O_2_, which is connected by a lead to external instrumentation. Hypoxic tumor areas are more resistant to radio- and chemotherapy; therefore identifying them by means of an implantable sensor may enable these regions to be targeted more efficiently. However, the use of this sensor may not be restricted to cancer patients and may have additional applications in which monitoring visceral surface tissue O_2_ tension ($\text{P}{\text{T}}_{{\text{o}}_{2}}$) is clinically important. One potential situation where assessment of tissue perfusion would be advantageous is in the perioperative management of patients undergoing intestinal surgery.

Intestinal resection and anastomosis is a commonly performed abdominal operation for a variety of benign and malignant diseases. The procedure involves removal (resection) of a segment of intestine with the subsequent rejoining (anastomosis) of the intestinal ends in a manner that optimizes healing and restores mural and luminal integrity. However, if the anastomosis fails to heal and a leak develops, patients suffer a significant increase in morbidity (17, 23, 28) and mortality (9). Leak rates can be as high as 24% for surgery performed in the distal rectum; however, overall leak incidence is generally accepted to be ~6–7% (6, 21). The etiology of an anastomotic leak is multifactorial; however, blood flow disruption, leading to compromised tissue perfusion and decreased oxygenation is known to affect anastomotic healing through the development of perianastomotic necrosis (38). Surgeons typically assess intraoperative intestinal perfusion through macroscopic tissue appraisal; however, this subjective technique is unable to predict the risk of a leak occurring. It is suggested that objective measurements of tissue perfusion at the anastomosis can aid the identification of anastomotic sites at increased risk of leakage (24). Methods used to assess tissue perfusion (visible light and near-infrared spectroscopy and laser fluorescence angiography) (13, 19) and oxygen tensions (scanning laser Doppler flowmetry and wireless handheld pulse oximeters) (7, 38) have been shown to help predict the occurrence of a leak. Visible light spectroscopy has also been shown to be compatible with endoscopic procedures (5, 13, 25). Currently, there is one clinical trial under way assessing whether the intraoperative measurement of anastomotic perfusion by use of a fluorescent dye (indocyanine green) and near-infrared laparoscopy can minimize the occurrence of leaks compared with conventional white-light laparoscopy (3). Preclinical (26, 39) and clinical (40, 41) studies have also shown that Clark O_2_ sensors cannot only measure intestinal $\text{P}{\text{T}}_{{\text{o}}_{2}}$, but also predict the occurrence of an anastomotic leak. These studies used O_2_ sensors consisting of a platinum cathode and silver anode, which were used to measure intraoperative intestinal $\text{P}{\text{T}}_{{\text{o}}_{2}}$. Intraoperative measurements were only possible due to the large size of the electrodes used in the preclinical studies (62 × 7 mm, intestinal contact surface 2 mm) (39) and because handheld devices were used in both clinical studies.

Unfortunately, none of these techniques are used routinely by clinicians and are only applicable to intraoperative use; they cannot be left in situ as a means of real-time, continuous, postoperative monitoring. The development of a miniaturized implantable device for the continuous intra- and postoperative monitoring of intestinal $\text{P}{\text{T}}_{{\text{o}}_{2}}$ could provide an additional means by which anastomotic healing could be monitored, thereby providing a means by which perioperative patient treatment could be optimized. In this study, we describe the development and use of an anesthetized rat intestinal ischemic and hypoxic model for validation of the IMPACT O_2_ sensor to provide real-time measurement of intestinal $\text{P}{\text{T}}_{{\text{o}}_{2}}$.

## MATERIALS AND METHODS

Studies were undertaken under a UK Home Office Project License in accordance with the Animals (Scientific Procedures) Act 1986 and with approval from the University College London Animal Ethics Committee. Male Wistar rats (Charles River Laboratories, Margate, UK) of ~400 g body weight were used in all experiments (*n* ≥ 4/treatment group). Before instrumentation, the rats were acclimatized for at least 1 wk and housed in cages of 4 on an alternating 12:12-h light-dark cycle with ad libitum access to food and water.

**Fig. 1.**
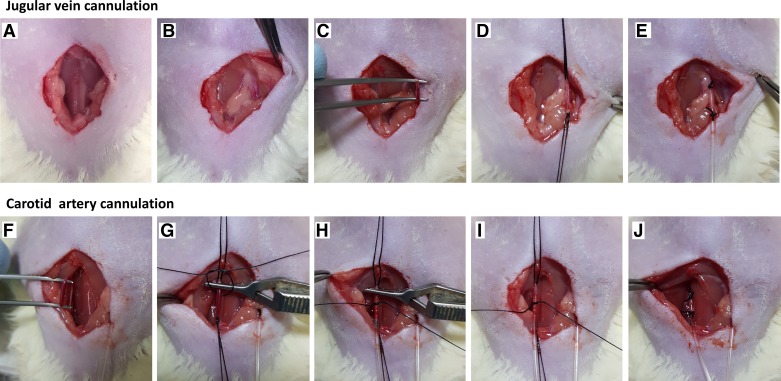
Intraoperative photographs depicting placement of central venous (jugular) and arterial (carotid) lines. *A*: a 2-cm ventral cervical skin incision is made. *B* and *C*: cervical dissection is performed for identification and mobilization of the right jugular vein. *D*: one untied suture is placed distally around the jugular vein and a second proximally and tied securely, leaving ~1 cm of vein between the 2 sutures. *E*: a venotomy is created just distal to the proximal suture; the cannula is inserted and advanced into the vein ~2–3 cm before tying the distal suture around the vein and cannula. *F*: cervical dissection is performed for identification of the left carotid artery and its isolation from the vagus nerve. *G*: one untied suture is placed distally around the carotid artery and a second proximally and tied securely, leaving ~1.5 cm of artery between the 2 sutures. A small clamp is added just proximal to the distal suture before placing a third untied suture. *H*: an arteriotomy is created between the 2 sutures, and the cannula is inserted and advanced up to the clamp. *I*: a third suture is tied loosely over the cannula, and the clamp is removed. *J*: the cannula is advanced a further 2 cm before tying the distal suture around the artery and cannula. Both vessels were cannulated using 0.96-mm outside diameter PVC tubing (Scientific Commodities, Lake Havasu City, AZ) and secured in place with 5-0 silk (Ethicon).

Rats were anesthetized using isoflurane (Abbott, Maidenhead, UK) delivered in room air (5% for induction, 2% for surgical procedures, and 1.5% for maintenance). Rats were placed on a heated mat to maintain rectal temperature between 36.5 and 37.5°C, which was continuously monitored using a temperature sensor (Harvard Apparatus, Cambridge, UK) inserted into the rectum. Placement of arterial and venous lines was performed as reported previously (10) (Fig. 1). The arterial line was subsequently connected to a pressure transducer system (Powerlab and Chart 5 acquisition software; AD Instruments, Chalgrove, UK) for continuous monitoring and recording of mean arterial blood pressure and heart rate. The arterial line was also used for intermittent blood sampling; when required, 0.1 ml of arterial blood was taken into heparinized capillary tubes for blood gas analysis (ABL90 Analyzer; Radiometer, Copenhagen, Denmark). The venous line was used for administration of fluids; all animals received a continuous infusion of 10–20 ml·kg^−1^·h^−1^ compound sodium lactate (Baxter, Thetford, UK) to maintain mean arterial blood pressure at ~100 mmHg. A tracheostomy was created using 2.08-mm external diameter polythene tubing (Portex, Hythe, UK); this was attached to a T-piece to secure the airway, maintain anesthesia, and allow alterations in the inspired fractional O_2_ concentration ($\text{F}{\text{I}}_{{\text{o}}_{\text{2}}}$).

**Fig. 2.**
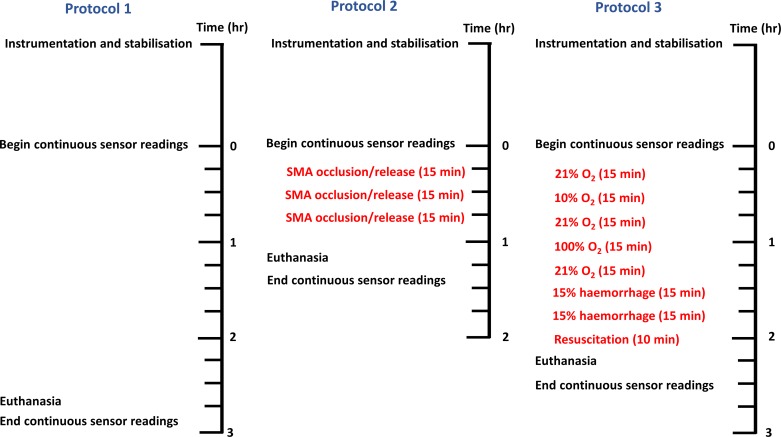
Outline of experimental protocols and interventions. *Protocol 1*: control animals with no interventions. *Protocol 2*: superior mesenteric artery (SMA) occlusion. *Protocol 3*: alterations in inspired fractional O_2_ concentration ($\text{F}{\text{I}}_{{\text{o}}_{\text{2}}}$), hemorrhage, and autotransfusion (resuscitation). Instrumentation encompassed anesthesia induction, surgical preparation, placement of arterial and venous lines, and celiotomy. The sensor was placed on the serosal surface of the small intestine approximately halfway through the stabilization period. Continuous sensor readings were commenced following achievement of stable baseline physiological variables and continued for 15 min following confirmation of death post-euthanasia.

**Fig. 3.**
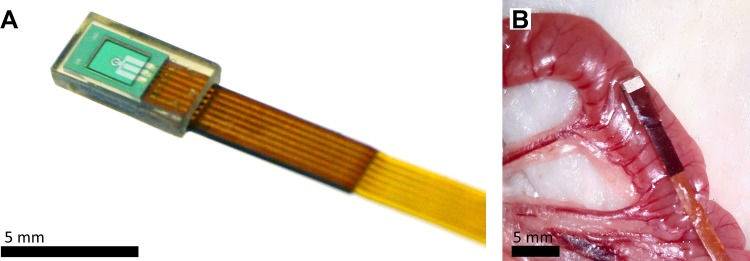
Sensor picture and intraoperative photographs depicting intestinal placement of the sensor. *A*: macroscopic image of the Implantable Microsystems for Personalized Anti-Cancer Therapy (IMPACT) miniaturized O_2_ sensor. *B*: intraoperative sensor placement on the serosal surface of a section of small intestine.

One control group (to assess sensor longevity and baseline drift) and two treatment groups (either superior mesenteric artery occlusion or manipulation of $\text{F}{\text{I}}_{{\text{o}}_{\text{2}}}$ and circulating blood volume) were used to assess whether the sensor could detect changes in intestinal oxygenation following local or systemic cardiorespiratory insults (Fig. 2). A ventral midline celiotomy was performed on all animals, extending from the pubis to the manubrium, and a temporary cystotomy was created by making a small stab incision at the apex of the bladder, which was then cannulated with 1.57-mm external diameter polythene tubing (Portex) and sutured in place. To allow access to the abdominal vasculature and placement of the sensor, the cecum and small intestine were exteriorized to the left side on the animal, wrapped in cling film (to reduce evaporative fluid and convective heat loss) and kept moist with regular topical applications of saline. The O_2_ sensor was placed on the antimesenteric serosal surface of a proximal section of small intestine. The active area of the sensor was kept moist and placed at the midpoint between small serosal blood vessels (Fig. 3). The sensor lead was temporarily attached to the serosal surface of the bowel with tissue adhesive (Vetbond; 3M, Bracknell, UK). After instrumentation was completed, all animals were allowed a period of stabilization of at least 30 min to achieve stable baseline physiological variables. Continuous sensor readings were made throughout each experiment, commencing after the initial stabilization period and continuing for 15 min following confirmation of death posteuthanasia. All animals were euthanized with intravenous pentobarbital sodium (Pentoject; Animalcare, York, UK).

### 

#### Oxygen sensor recordings.

The IMPACT O_2_ sensor contains a complete miniaturized three-electrode cell with platinum working and counter-electrodes and a silver/silver chloride reference electrode. Nafion was used to cover the electrode surfaces to reduce electrode biofouling and improve sensor lifetime. Electrodes were fabricated on silicon wafers by using conventional microfabrication techniques, and diced into 2.0  × 3.0-mm chips, each of which carried a single sensor. Chips were wire bonded to a 1.7  × 200-mm long lead, and the chip was sealed in biocompatible epoxy resin, giving an overall sensor size of ~2.8  × 5.0  × 1.4 mm (width × length × height), with a clear window over the central active area to permit sensor access to the tissue environment. Following intestinal placement/implantation, the sensor lead was connected to an EmStat3 Blue potentiostat (PalmSens, Houten, The Netherlands). Periodic O_2_ measurements were performed using chronoamperometry at −0.5 V (vs. the on-chip Ag/AgCl reference electrode) for 20 s, followed by a rest period of 20 s, repeated continuously for the duration of the experiment. The working electrode current over the final 5 s of each chronoamperometry recording was averaged using MATLAB R2017a (MathWorks, Natick, MA) to derive the mean current. We have previously shown that the measured current magnitude is proportional to local Po_2_ at the sensor surface in saline. However, since there may be small environmental differences between saline and tissue that could affect the slope of the sensor response in vivo, we chose to directly quantify its electrode current in this study.

#### Protocol 1: control group.

The control group of animals consisted only of continuous sensor readings taken for 165 min. These animals were sham operated, receiving only a ventral midline celiotomy (as described in the previous section) for sensor placement, with no superior mesenteric artery (SMA) occlusions or manipulations of $\text{F}{\text{I}}_{{\text{o}}_{\text{2}}}$ or circulating blood volume being performed (i.e. no physiological challenges were performed on these instrumented animals). Arterial blood samples were obtained immediately before the start of continuous sensor readings, every hour thereafter, and then immediately before euthanasia.

#### Protocol 2: intestinal tissue responses to temporary superior mesenteric artery occlusion.

For temporary SMA occlusion, the vessel was first identified originating from the left side of the aorta at the level of the right renal vein and joining the caudal vena cava. The SMA was dissected free from surrounding soft tissue and its associated vein as close to its aortic origin as possible. A loose suture of 5-0 silk (Ethicon, Livingston, UK) was placed round the artery for ease of location and to allow gentle traction to aid its temporary and reversible mechanical occlusion. After its isolation, the vessel was repeatedly occluded with the use of a hemostatic clamp (Fine Science Tools, Linton, UK). Each occlusion was performed for 5 min with a 10-min reperfusion period between occlusions. Vessel occlusion was confirmed by loss of pulsations in the mesenteric arteries and intestinal pallor. A hyperemic flush and the presence of mesenteric arterial pulsations were evident following removal of the hemostatic clamp. Arterial blood samples were obtained immediately before the start of continuous sensor readings and just before euthanasia.

#### Protocol 3: intestinal tissue responses to alterations in inspired oxygen concentrations, progressive hemorrhage and autotransfusion.

$\text{F}{\text{I}}_{{\text{o}}_{\text{2}}}$ (1.0, 0.21, 0.1) was varied at 15-min intervals and delivered through a flowmeter via the isoflurane vaporizer to maintain adequate anesthesia. The protocol for varying $\text{F}{\text{I}}_{{\text{o}}_{\text{2}}}$ was 0.21 for 15 min, 0.1 for 15 min (inducing hypoxemia), 0.21 for 15 min, and 1.0 for 15 min (inducing hyperoxemia) before returning to 0.21 for 15 min. Arterial blood samples were taken at the end of each $\text{F}{\text{I}}_{{\text{o}}_{\text{2}}}$ alteration.

After completion of the $\text{F}{\text{I}}_{{\text{o}}_{\text{2}}}$ modifications, progressive hemorrhage was achieved by withdrawing 15% of the animal's circulating blood volume (estimated based on 70 ml/kg body wt) over 5 min. The animal was allowed to stabilize for 10 min before a further 15% circulating blood volume was removed. The exsanguinated blood was collected in a heparinized syringe and maintained at 37°C. Autotransfusion was performed by administering the exsanguinated blood intravenously to the animal over 10 min. Arterial blood samples were taken at the end of each hemorrhage procedure and immediately before euthanasia.

#### Statistical analysis.

Data were confirmed as normally distributed using the Shapiro-Wilk test and subsequently analyzed with parametric tests; one-way ANOVA with Holm-Šídák multiple comparisons test was used to test for differences between groups; *P* values <0.05 were deemed statistically significant. Data are expressed as means ± SE. Statistical analyses were performed using Prism 7 (GraphPad Software, San Diego, CA).

## RESULTS

### 

#### Sensor readings remained stable in control animals.

**Fig. 4.**
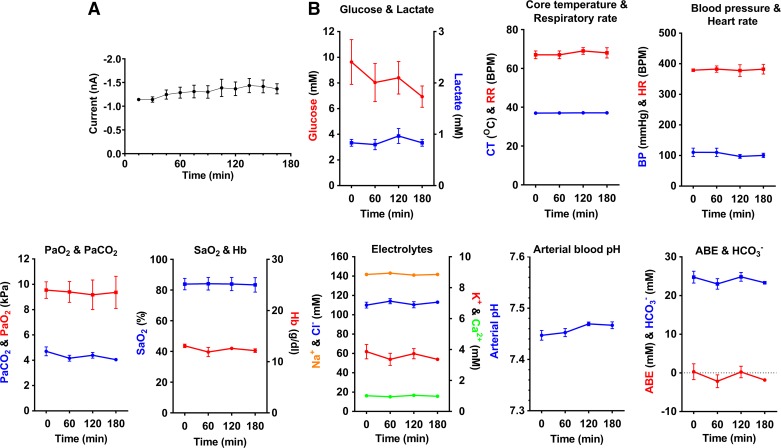
Sensor readings and physiological data obtained from control animals (*protocol 1*). *A*: current generated at the working electrode of the sensor during continuous sensor readings over a period of 165 min; results show mean current readings from the final 5 min of each 15-min period (one-way ANOVA with Holm-Šídák multiple comparisons test; data expressed as means ± SE, *n* = 3 rats, *P* = 0.7244). *B*: physiological data obtained throughout the experimental period. CT, core temperature; RR, respiratory rate; HR, heart rate; BP, blood pressure; Hb, hemoglobin; ABE, arterial base excess; $\text{S}{\text{a}}_{{\text{O}}_{2}}$, Hb O_2_ saturation; $\text{P}{\text{a}}_{\text{O}}{}_{{}_{2}}$, arterial O_2_ partial pressure; $\text{P}{\text{a}}_{\text{C}\text{O}}{}_{{}_{2}}$, arterial CO_2_ partial pressure; $\text{H}\text{C}{\text{O}}_{3}^{-}$, bicarbonate.

For assessment of sensor stability, we performed continuous sensor readings in instrumented rats that were not subjected to any physiological interventions (*n* = 3). These control experiments were of a duration that encompassed the longest experimental protocol. The negative currents generated in the O_2_ sensors are proportional with $\text{P}{\text{T}}_{{\text{o}}_{2}}$, with more negative values indicating greater O_2_ partial pressures. The measured currents were stable over 165 min, with mean values ranging from −1.14 ± 0.03 nA at 15 min to −1.44 ± 0.15 nA at 135 min (*P* = 0.7244; Fig. 4*A*).

To assess the stability of the rats during anesthesia, physiological data, including core temperature (CT), heart rate (HR), respiratory rate (RR), and mean arterial blood pressure (MAP), and intermittent blood gas and biochemistry samples were evaluated. All physiological data, blood gas analysis and electrolytes remained stable; only blood glucose levels decreased slightly toward the end of the procedure. Although this reduction was not statistically significant (*P* = 0.9304), it is consistent with the fasting state and metabolic demands of the rat during anesthesia (Fig. 4*B*). The physiological data obtained, combined with arterial blood analysis, provided evidence that the rats were able to cope with the physiological demands from general anesthesia for the duration of our experimental protocols. These results are in accord with other previous studies (11).

#### Oxygen current generated by the sensor rapidly decreased following occlusion of the SMA and was reversible upon reinstatement of blood flow.

**Fig. 5.**
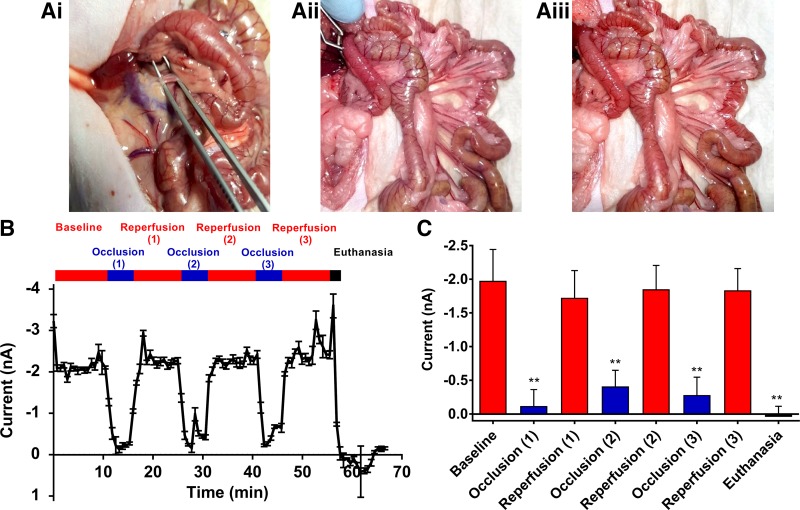
Intraoperative photographs and sensor readings during temporary superior mesenteric artery (SMA) occlusion (*protocol 2*). *Ai*: identification of SMA originating from the left side of the aorta at the level of the right renal vein joining the caudal vena cava; the vessel is robust and can withstand the manipulation required for isolation from its surrounding tissue and associated vein. *Aii*: a Bulldog clamp was placed across the SMA for temporary occlusion. Note the intestinal pallor that was immediately evident upon occlusion. *Aiii*: after removal of the Bulldog clamp and reinstatement of blood flow, a hyperemic flush was evident to the intestines. *B*: representative graph showing current generated at the working electrode of the sensor during continuous sensor readings over a period of 70 min. Three cycles of 5 min occlusion followed by 10 min of reperfusion were performed in each animal before euthanasia. *C*: combined analysis comparing current generated at the working electrode of the sensor during periods of SMA occlusion and intestinal reperfusion; results show mean current readings of the final 3 min of each occlusion period and final 5 min of each reperfusion period (one-way ANOVA with Holm-Šídák multiple comparisons test; data expressed as means ± SE, *n* = 4 rats; **P ≤ 0.01).

To assess whether the sensor could detect changes in intestinal oxygenation through the generation of localized tissue ischemia, we performed three cycles of temporary SMA occlusion followed by release in each rat (*n* = 4; Fig. 5*A*). After each occlusion, there was an almost immediate fall in sensor current from nonoccluded readings of −1.85 ± 0.05 nA to occluded readings of −0.27 ± 0.08 nA. The decrease in current that occurred during each occlusion was statistically significant compared with current values pre- and postocclusion (*P* = 0.0082). The decrease in current during the occlusion quickly rose back to nonoccluded current levels following reinstatement of blood flow (Fig. 5*B* and Table 1). After euthanasia of the animal, sensor readings fell to the lowest recorded, measuring 0.04 ± 0.16 nA; this decrease was statistically significant compared with nonoccluded SMA currents (*P* = 0.0025; Fig. 5*C*). These results showed that the sensor could quickly, reliably, and reproducibly detect transient intestinal ischemia.

**Table 1.** Current readings generated during each superior mesenteric artery occlusion and reperfusion period and euthanasia

	Baseline	Occlusion (1)	Reperfusion (1)	Occlusion (2)	Reperfusion (2)	Occlusion (3)	Reperfusion (3)	Euthanasia
Current, nA	−1.98 ± 0.47	−0.12 ± 0.25	−1.73 ± 0.40	−0.41 ± 0.24	−1.85 ± 0.36	−0.28 ± 0.27	−1.84 ± 0.32	0.04 ± 0.16

Values are means ± SE; *n* = 4 rats. Numbers in parentheses correspond to each specific occlusion/reperfusion challenge detailed in Fig. 5.

#### Oxygen current generated by the sensor responded rapidly to alterations in inspired oxygen concentrations.

**Fig. 6.**
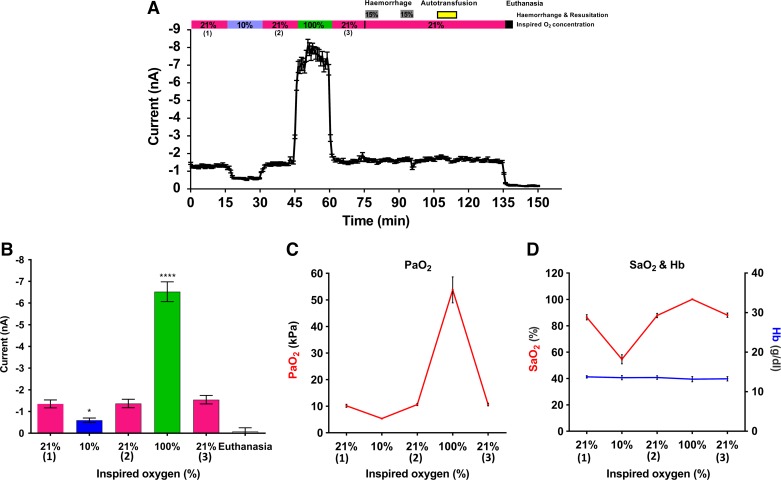
Sensor readings and physiological data obtained from assessing intestinal tissue oxygenation following changes in inspired O_2_ concentrations (*protocol 3A*). *A*: representative graph showing current generated at the working electrode of the sensor during continuous sensor readings over a period of 150 min. Fifteen-minute blocks of each inspired fractional O_2_ concentration ($\text{F}{\text{I}}_{{\text{o}}_{\text{2}}}$) challenge were performed before beginning removal of 30% of circulating blood volume. *B*: combined analysis comparing current generated at the working electrode of the sensor during each $\text{F}{\text{I}}_{{\text{o}}_{\text{2}}}$ challenge; results show mean current readings of the final 5 min of each 15-min period (one-way ANOVA with Holm-Šídák multiple comparisons test; data are expressed as means ± SE; *n* = 5 rats; ****P ≤ 0.0001, *P ≤ 0.05). *C* and *D*: arterial O_2_ partial pressure ($\text{P}{\text{a}}_{\text{O}}{}_{{}_{2}}$), total hemoglobin (Hb), and Hb O_2_ saturation ($\text{S}{\text{a}}_{{\text{O}}_{2}}$) measured at end of each $\text{F}{\text{I}}_{{\text{o}}_{\text{2}}}$ challenge.

To assess whether the sensor could detect changes in intestinal oxygenation through alterations in $\text{F}{\text{I}}_{{\text{o}}_{\text{2}}}$, we performed sequential inspired O_2_ challenges varying from 0.1 to 1.0 (*n* = 5). With a $\text{F}{\text{I}}_{{\text{o}}_{\text{2}}}$ of 0.21 (breathing room air), the mean sensor current was −1.35 ± 0.06 nA. When $\text{F}{\text{I}}_{{\text{o}}_{\text{2}}}$ was reduced to 0.1, the sensor current decreased by more than one-half, to −0.60 ± 0.10 nA. This decrease was statistically significant compared with currents generated when the animals were breathing room air (*P* = 0.0396). The decrease in current occurred over 1–2 min and was maintained throughout the hypoxemic challenge. After restoration of $\text{F}{\text{I}}_{{\text{o}}_{\text{2}}}$ to 0.21, the current returned to baseline levels over a period of 1–2 min. Upon increasing inspired $\text{F}{\text{I}}_{{\text{o}}_{\text{2}}}$ to 1.0, the sensor current rose to −6.53 ± 0.46 nA, which was statistically significant compared with currents generated with a $\text{F}{\text{I}}_{{\text{o}}_{\text{2}}}$ of 0.21 (*P* < 0.0001). This increase in current was again maintained throughout the $\text{F}{\text{I}}_{{\text{o}}_{\text{2}}}$ challenge. Returning $\text{F}{\text{I}}_{{\text{o}}_{\text{2}}}$ back to room air saw sensor currents return to baseline levels. After euthanasia of the animal, sensor readings fell to the lowest recorded, measuring −0.08 ± 0.17 nA (Fig. 6*A*). These results showed that the sensor could quickly, reliably, and reproducibly detect changes in intestinal oxygenation following alterations in $\text{F}{\text{I}}_{{\text{o}}_{\text{2}}}$ (Fig. 6*B* and Table 2).

**Table 2.** Current readings generated during each $FI_{O_{2}}$ challenge and euthanasia

	21% (1)	10%	21% (2)	100%	21% (3)	Euthanasia
Current, nA	−1.35 ± 0.18	−0.60 ± 0.10	−1.37 ± 0.12	−6.53 ± 0.46	−1.55 ± 0.20	−0.08 ± 0.17

Values are means ± SE; *n* = 5 rats. Numbers in parentheses correspond to each specific inspired fractional O_2_ concentration ($\text{F}{\text{I}}_{{\text{o}}_{\text{2}}}$) challenge detailed in Fig. 6.

Blood gas analysis at the end of each $\text{F}{\text{I}}_{{\text{o}}_{\text{2}}}$ challenge showed the expected physiological alterations in arterial O_2_ partial pressure ($\text{P}{\text{a}}_{\text{O}}{}_{{}_{2}}$) and hemoglobin O_2_ saturation ($\text{S}{\text{a}}_{{\text{O}}_{2}}$). Both variables decreased significantly at a $\text{F}{\text{I}}_{{\text{o}}_{\text{2}}}$ of 0.1, increased significantly at a $\text{F}{\text{I}}_{{\text{o}}_{\text{2}}}$ of 1.0 (more prominent with $\text{P}{\text{a}}_{\text{O}}{}_{{}_{2}}$), and returned to baseline levels at 0.21. Total hemoglobin (Hb) concentration remained constant (Fig. 6, *C* and *D*).

#### No change in sensor current was identified following progressive hemorrhage.

**Fig. 7.**
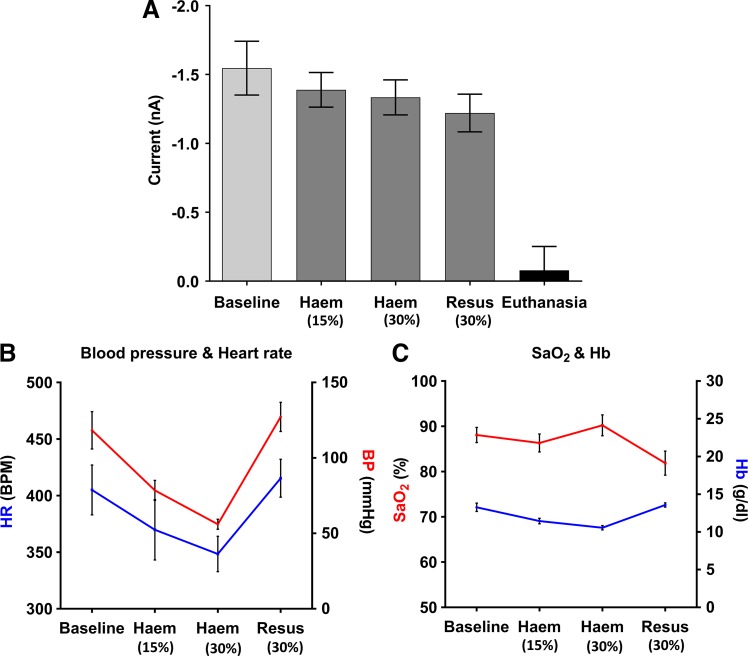
Sensor readings and physiological data obtained from assessing intestinal tissue oxygenation following progressive hemorrhage and autotransfusion (*protocol 3B*). *A*: combined analysis comparing current generated at the working electrode of the sensor during the preceding 15 min baseline period at 21% O_2_ and subsequent 2 cycles of removal of 15% of circulating blood volume followed by autotransfusion; results show mean current readings of the final 5 min of each period (one-way ANOVA with Holm-Šídák’s multiple comparisons test; data are expressed as means ± SE, *n* = 5 rats). *B* and *C*: mean arterial blood pressure, heart rate, total hemoglobin, and hemoglobin O_2_ saturation measured at end of each intervention period. Haem, hemorrhage; Resus, autotransfusion resuscitation.

After completion of $\text{F}{\text{I}}_{{\text{o}}_{\text{2}}}$ challenges, we assessed the ability of the sensor to detect changes in intestinal $\text{P}{\text{T}}_{{\text{o}}_{2}}$ following progressive withdrawal of 30% circulating blood volume (performed in 2 × 15% removal periods, *n* = 5; Fig. 7*A*). Physiological data obtained 10 min after the end of each hemorrhage procedure showed the expected physiological response occurring from acute hemorrhage: HR, MAP, and Hb concentration all showed a moderate decrease after 15% removal, which was even more pronounced after 30%; HR decreased from 405 ± 22 to 348 ± 16 beats/min, MAP decreased from 118 ± 12 to 56 ± 3 mmHg, and Hb decreased from 13.3 ± 0.6 to 11.4 ± 0.4 g/dl. All of these physiological parameters returned to baseline levels following autotransfusion (Fig. 7, *B* and *C*). $\text{S}{\text{a}}_{{\text{O}}_{2}}$ remained constant throughout each hemorrhage procedure.

Despite these expected physiological responses to acute hemorrhage, only a small and nonsignificant decrease in sensor current was observed. Baseline measurements were −1.55 ± 0.20 nA; however, following 15% and 30% hemorrhage, currents decreased only to −1.39 ± 0.13 and −1.33 ± 0.13 nA, respectively. After autotransfusion, no statistically significant changes in sensor current occurred, measuring −1.22 ± 0.14 nA at the end of the transfusion (Table 3).

**Table 3.** Current readings generated during 21% $FI_{o_{2}}$, each hemorrhage procedure, and autotransfusion

	Baseline	15% Hemorrhage	30% Hemorrhage	Autotransfusion
Current, nA	−1.55 ± 0.20	−1.39 ± 0.13	−1.33 ± 0.13	−1.22 ± 0.14

Values are means ± SE; *n* = 5 rats. $\text{F}{\text{I}}_{{\text{o}}_{\text{2}}}$, inspired fractional O_2_ concentration.

## DISCUSSION

For validation and proof of concept that the IMPACT O_2_ sensor can provide real-time measurements of intestinal $\text{P}{\text{T}}_{{\text{o}}_{2}}$ we developed an in vivo intestinal ischemic (SMA occlusion) and hypoxic (changes in $\text{F}{\text{I}}_{{\text{o}}_{\text{2}}}$) model. Control experiments were first used to demonstrate that the rats could remain physiologically stable under general anesthesia and that the sensors provided stable measurements. These experiments also allowed us to rule out potential effects of the tissue environment on sensor stability; the design and packaging of the IMPACT O_2_ sensor is likely to have minimized this potential problem. The outer protective coating is made from Nafion, an ionomer made by the addition of sulfonic acid groups to Teflon. Nafion membranes are commonly used in a variety of implantable devices due to the selectivity of substances that diffuse through it. The pore size within the membrane is sufficiently small to exclude macromolecules such as proteins that may contaminate the electrode surface, while the presence of charged sulfonate groups leads to the electrostatic exclusion of small anions (34).

Our sensors were placed onto the intestinal serosal surface and secured with tissue glue. This technique avoided the use of constant manual pressure on the sensor, which could interfere with intestinal blood supply leading to alterations in $\text{P}{\text{T}}_{{\text{o}}_{2}}$ measurements (40). The tissue area from which the electrode measures $\text{P}{\text{T}}_{{\text{o}}_{2}}$ is related to electrode size, a matter that needs to be considered if Clark electrodes are used for clinical applications. The working electrode (where O_2_ is detected) of the IMPACT O_2_ sensor has a diameter of 50 µm. This small sensing area means the sensor will not significantly deplete tissue O_2_ levels while also allowing for accurate sensor placement to specific areas by the surgeon. Use of multiple sensors would provide $\text{P}{\text{T}}_{{\text{o}}_{2}}$ readings from a larger area, which is advantageous for anastomotic surgery, as sensors could be placed around the anastomotic circumference and at either side of the resection site, giving detailed perianastomotic $\text{P}{\text{T}}_{{\text{o}}_{2}}$ information. Such detailed information could not be achieved using a sensor with a large working area that gives only mean $\text{P}{\text{T}}_{{\text{o}}_{2}}$ readings from multiple capillary beds, as is likely the case in the preclinical and clinical studies using Clark electrodes described in the introduction.

The SMA is the principal arterial supply to a large part of the gastrointestinal tract (from the lower part of the duodenum through to the proximal two-thirds of the transverse colon and pancreas); its occlusion is the most direct way of causing intestinal ischemia. Various SMA occlusion techniques have been documented in previous studies for the investigation of ischemic intestinal insults, including ischemia-reperfusion injuries (14, 22), cytokine production, and bacteria/endotoxin translocation (16). In our study, we performed temporary SMA ligation close to its aortic origin to ensure that no collateral circulation from any of its branches (inferior pancreatico-duodenal, intestinal, ileocolic, and right and middle colic) could supply the region of small intestine of interest. Our results showed that within 1–2 min after SMA occlusion the current generated by the sensor fell to almost 0 nA, indicating a fully hypoxic state. This fall in current will be related to ischemia and utilization of the residual available O_2_ by the tissues. Reperfusion of the intestine led to both visual changes in the tissue (hyperemic flush and reoccurrence of mesenteric arterial pulsations) and a rapid rise in sensor current, indicating a return of normal oxygenation status to the intestine. This shows that the sensor reliably and reproducibly detects tissue hypoxia.

After it was confirmed that changes in intestinal $\text{P}{\text{T}}_{{\text{o}}_{2}}$ could be detected through ischemic insults, we determined whether alterations in blood oxygenation, through changes in $\text{F}{\text{I}}_{{\text{o}}_{\text{2}}}$, could be detected. Arterial blood gas analysis confirmed the expected physiological responses to $\text{F}{\text{I}}_{{\text{o}}_{\text{2}}}$ challenges. Hypoxemia caused a decrease in $\text{P}{\text{a}}_{\text{O}}{}_{{}_{2}}$ and $\text{S}{\text{a}}_{{\text{O}}_{2}}$, whereas hyperoxemia led to an increase in their levels. Sensor current readings mirrored these results, which changed in accordance with $\text{F}{\text{I}}_{{\text{o}}_{\text{2}}}$. Response times, as with ischemia, were rapid, with the sensor giving stable currents 1–2 min after a $\text{F}{\text{I}}_{{\text{o}}_{\text{2}}}$ change.

Although changes in intestinal $\text{P}{\text{T}}_{{\text{o}}_{2}}$ were detected though SMA occlusion and $\text{F}{\text{I}}_{{\text{o}}_{\text{2}}}$ alterations, no such changes were evident with acute hemorrhage. The reductions in HR and BP following 30% removal of circulating blood volume are consistent with increased vagal activity and reduced sympathetic input (Bezold-Jarish-like reflex), resulting in a decrease in total peripheral resistance. Only with more profound reductions in circulating blood volume is HR likely to increase during hemorrhage, representing a transition from reversible to irreversible shock (37). Although a slight decrease in Hb concentration was noted with 30% hemorrhage, $\text{S}{\text{a}}_{{\text{O}}_{2}}$ levels remained consistent. Stable $\text{S}{\text{a}}_{{\text{O}}_{2}}$ levels were likely due to the hemorrhage procedures being performed using an $\text{F}{\text{I}}_{{\text{o}}_{\text{2}}}$ of 0.21. This level of $\text{S}{\text{a}}_{{\text{O}}_{2}}$ in combination with a decrease in peripheral resistance may have been sufficient to maintain intestinal $\text{P}{\text{T}}_{{\text{o}}_{2}}$, which could explain why no change in sensor currents were observed.

One of the main advantages of our experiments over previous published studies is that they were designed not only to assess whether our sensor could detect a change in intestinal $\text{P}{\text{T}}_{{\text{o}}_{2}}$ following an initial physiological challenge but also whether sensor readings could revert to baseline levels upon removal of the challenge. Previous work has reported results mainly by using individual measurements at specific time points (26, 39–41); the continuous recording of data performed in this study provided an opportunity to measure $\text{P}{\text{T}}_{{\text{o}}_{2}}$ changes dynamically before, during, and after each challenge. Our results reproducibly showed that sensor currents returned to baseline levels following each challenge.

For any implantable medical device to gain clinical approval, it must undergo biocompatibility evaluation to assess biofunctionality (performance) and biosafety (local and systemic tissue responses and the absence of carcinogenesis, mutagenesis, and cytotoxicity) (4, 30, 36). After implantation, medical devices can lose functionality, largely due to biofouling (nonspecific protein adsorption) and the production of granulation and fibrous tissue around the implanted device (2). This process, known as the foreign body response (FBR), is composed of both acute and chronic inflammatory phases (2, 30, 31) and represents a clinical challenge in implantable technology design and development. The materials used in the manufacture of the IMPACT sensor have previously undergone extensive biocompatibility evaluation in both normal and diseased tissues, with results indicating that the materials are well tolerated with a minimal FBR elicited when implanted in vivo. These results have led to the use of these materials in a variety of medical devices (15). Although the FBR was not evaluated in this study, it is possible that acute inflammation could occur through either intestinal manipulation, placement of the sensor on the intestinal serosal surface, or through the use of tissue glue to secure the sensor in place. To minimize these potential effects, the intestine was handled as little as possible, and the glue was placed away from the sensor’s sensing area. Chronic inflammation was also not evaluated in this study, largely due to the short-term nature of the experiments; however, we anticipate that in a clinical situation the effects from chronic inflammation may not be a significant issue. Fibrous reactions that occur around implantable devices occur mainly during the chronic inflammatory phase (5–21 days post-implantation) (1); since the majority of anastomotic leaks occur within the first 8 days post-surgery (18), the use of a long-term implantable device is unlikely to be needed, and this situation would reduce concerns regarding the impact that chronic inflammation can have on sensor functionality.

One potential limitation to these experiments is that both the SMA occlusion technique and alterations in $\text{F}{\text{I}}_{{\text{o}}_{\text{2}}}$ (0.1 to 1.0) represent extreme experimental insults that may not accurately represent physiological ischemic and hypoxemic changes that occur following intestinal surgery. However, the primary aim of this study was to validate the ability of the IMPACT sensor to measure dynamic changes in intestinal $\text{P}{\text{T}}_{{\text{o}}_{2}}$. Our results provide justification to progress validation experiments into more complex large animal anastomotic leak models. Pigs are regarded as excellent gastrointestinal translational models, as they offer similarities in size, anatomy, and physiology to those of humans, and a porcine rectal anastomotic leak model has been described in the literature (43). Development of this porcine model could be performed for use in future experiments with the IMPACT O_2_ sensor. These next phases of experiments would investigate the sensor’s potential to monitor intestinal $\text{P}{\text{T}}_{{\text{o}}_{2}}$ in normal physiological conditions and in those situations associated with an anastomotic leak and assess the suitability of the sensors for surgical implantation. Medium- to long-term duration experiments would also allow the FBR and its effects on sensor functionality to be investigated. The use of this model would also allow evaluation of the feasibility of placing multiple sensors not only at the anastomotic site but also at multiple sites along the gastrointestinal tract. These studies will elucidate the potential of the sensors to be used in human patients for the early detection of anastomotic leakage and the hypoperfusion that may precede it. Experiments will also provide clinical, hematological, and biochemical indicators of anastomotic leakage and the development of septic peritonitis.

For the IMPACT O_2_ sensors to be used clinically for resection and anastomotic applications, investigations will need to be carried out to determine how these sensors could be placed intraoperatively, at the anastomotic site or at multiple sites along the gastrointestinal tract so that they remain in situ and are able to measure postoperative intestinal $\text{P}{\text{T}}_{{\text{o}}_{2}}$. Potential solutions would include their use akin to a surgical drain or through sensor incorporation into surgical staples. Drains are used commonly in anastomotic surgery (20, 33, 35) and are not associated with any significant complications. Placed in the region of the anastomotic site, the drains exit the abdominal cavity through a skin incision and are then connected to external drainage bags and are easily removed when no longer required. Drains have been used to obtain postoperative peritoneal samples from anastomotic sites for measuring bacterial colonization, cytokines (12), and metabolites (32) that might serve as indicators of early anastomotic leakage. We propose that designing the IMPACT O_2_ sensor in a similar manner to a surgical drain would allow easy clinical use. The sensors, along with an electrical lead (similar in design to the current IMPACT O_2_ sensor and lead wire), could be positioned on the intestine next to the anastomosis site at the time of surgery. The electrical lead would then pass out through the skin like that of a surgical drain, allowing the sensor to be connected to the end-of-bed monitor, providing postoperative real-time data on perianastomotic intestinal $\text{P}{\text{T}}_{{\text{o}}_{2}}$ levels. The sensors could then be removed in the same way as a surgical drain when no longer required. The advantage of this approach would be that the IMPACT O_2_ sensor would not require significant further development. Incorporation of IMPACT O_2_ sensors into staples would have the advantage that perianastomotic $\text{P}{\text{T}}_{{\text{o}}_{2}}$ could be measured around the entire circumference of the intestine, thus providing detailed spatial information. However, further technological development of the sensor through miniaturization and the development of wireless technology would be required to achieve sensor integration into surgical staples. If these engineering advancements could be achieved, it would provide opportunities for placing multiple sensors throughout the gastrointestinal tract, and this concept could be utilized for measuring mucosal $\text{P}{\text{T}}_{{\text{o}}_{2}}$ for other intestinal conditions where microenvironmental mucosal hypoxia can influence disease outcomes such as chronic intestinal inflammation (42).

In conclusion, using our developed rodent model of small intestinal ischemia and hypoxemia, we successfully demonstrated that the IMPACT O_2_ sensor can detect dynamic changes in intestinal oxygenation through ischemic insults, alterations in $\text{F}{\text{I}}_{{\text{o}}_{\text{2}}}$, and euthanasia. These miniaturized sensors have significant advantages over Clark O_2_ sensors previously used in the literature, as they have the potential to be used during surgery and be left in situ following surgery to provide postoperative, minimally invasive, continuous, real-time data regarding transitory changes in tissue $\text{P}{\text{T}}_{{\text{o}}_{2}}$. This novel method of monitoring tissue perfusion could, for example, detect early perianastomotic tissue hypoxia and identify patients that might benefit from treatments to improve local oxygenation and prevent a leak. The sensor’s capability to detect dynamic intestinal oxygenation changes could also be used to assess treatment responses and, when combined with information gained from conventional monitoring systems, would help provide a more accurate assessment of the patient’s condition. We expect that this type of visceral surface oximetry will prove clinically useful in the evaluation of bowel perfusion and may also be a powerful tool in many other clinical and research applications where measurement of organ perfusion is required, for example in transplant surgery, chronic intestinal ischemia and inflammation, plastic surgery, and brain injury.

## GRANTS

This work was supported by funding from the UK Engineering and Physical Sciences Research Council, through the IMPACT program grant (EP/K-34510/1) and by a project grant from Bowel and Cancer Research UK.

## DISCLOSURES

No conflicts of interest, financial or otherwise, are declared by the author(s).

## AUTHOR CONTRIBUTIONS

M.E.G., J.R.K.M., A.F.M., D.A., A.D., M.S., and M.A.P. conceived and designed research; M.E.G. and J.R.K.M. performed experiments; J.R.K.M., C.D., E.O., and A.T. fabricated the sensors; M.E.G. and J.R.K.M. analyzed data; M.E.G., J.R.K.M., and M.A.P. interpreted results of experiments; M.E.G., J.R.K.M., and J.M. prepared figures; M.E.G., J.R.K.M., and J.M. drafted manuscript; M.E.G., J.R.K.M., C.D., E.O.B., J.M., A.T., I.H.K., A.F.M., D.A., A.D., M.S., and M.A.P. edited and revised manuscript; M.E.G., J.R.K.M., C.D., E.O.B., J.M., A.T., I.H.K., A.F.M., D.A., A.D., M.S., and M.A.P. approved final version of manuscript.
